# Why does scabies persist despite effective treatment? Coordination failure, Nash equilibrium, and replicator dynamics

**DOI:** 10.3389/fpubh.2026.1865428

**Published:** 2026-09-16

**Authors:** Miryam Kerner

**Affiliations:** 1 The Ruth and Bruce Rapoport Faculty of Medicine, Technion - Israel Institute of Technology, Haifa, Israel; 2 Kaplan Medical Center, Rehovot, Israel

**Keywords:** coordination failure, mass drug administration, Nash equilibrium, reinfection, scabies, treatment compliance

## Abstract

**Introduction:**

Scabies remains a persistent global public health problem despite the availability of effective treatments, including topical permethrin, benzyl benzoate, crotamiton, and systemic ivermectin. This paper develops a formal economic-epidemiological framework using game theory to explain persistence through coordination failure and Nash equilibrium.

**Methods:**

Household treatment decisions are modeled as a coordination game under imperfect information, treatment costs, and reinfection externalities. The analysis is further extended using an evolutionary replicator dynamics framework to examine how parameter conditions determine equilibrium stability and long-run behavioral outcomes. To illustrate the theoretical framework, I analyzed global data linking scabies disability-adjusted life years (DALYs) to household washing machine coverage.

**Results:**

The analytical results demonstrate that individually rational behavior can lead to inefficient equilibria, resulting in recurrent infections and elevated societal costs. The empirical analysis reveals a statistically significant negative association between scabies DALYs and household washing machine coverage that is consistent across multiple yearly specifications and stable regional patterns of household washing machine coverage.

**Discussion:**

The empirical findings should be interpreted as correlational and illustrative rather than causal, since the empirical analysis does not control for the broader socioeconomic and institutional determinants of scabies burden. Nevertheless, the results are consistent with the theoretical framework and highlight the potential role of infrastructure-based hygiene interventions, in combination with mass drug administration and health education, in aligning individual incentives with socially optimal outcomes. Sustainable scabies control requires integrated economic, epidemiological, and behavioral strategies.

## 1 Introduction

Scabies, caused by the mite Sarcoptes scabiei var. hominis, is a neglected tropical disease with a substantial global burden (1, 2). Current estimates suggest that approximately 200–206 million individuals are infected at any given time, with more than 600 million new cases occurring annually. The disease contributes to approximately 5 million disability-adjusted life years (DALYs), reflecting both direct morbidity of very severe itching, especially at night accompanied by a rash, and secondary complications such as bacterial superinfection, post-streptococcal glomerulonephritis, and rheumatic heart disease (3–5). It often co-occurs with pyoderma and tinea infections, especially in pediatric populations (6). Skin disorders contribute substantially to global morbidity (7).

The epidemiology of scabies varies considerably across countries and regions. Scabies prevalence remains high in low-resource settings (8, 9). In low- and middle-income countries, particularly in tropical settings, the prevalence is often extremely high. In Papua New Guinea and Fiji, community prevalence can exceed 25%−30%, especially among children [(10, 11), based on national survey data in Fiji]. In Indonesia and parts of Sub-Saharan Africa, the prevalence typically ranges between 10% and 20% (12). In contrast, high-income countries such as the United Kingdom, Germany, and Israel report lower baseline prevalence—generally below 1%−2%—yet have experienced rising incidence and periodic outbreaks in institutional settings such as nursing homes, prisons, and schools (2). In Australia, scabies remains endemic in some Indigenous communities, with prevalence exceeding 20% in certain populations (12).

These patterns indicate that while economic development mitigates prevalence, it does not eliminate disease (12). Structural factors such as overcrowding, access to healthcare, and institutional transmission dynamics remain central (13). Importantly, scabies spreads primarily through prolonged skin-to-skin contact, making the household the key unit of transmission (1).

Effective scabies control requires the simultaneous treatment of all close contacts, including asymptomatic individuals, to prevent ongoing transmission and reinfestation (14, 15). Failure to treat—even by a single individual or through incomplete adherence—can sustain transmission within households and lead to reinfection cycles, as untreated contacts act as a continuing reservoir of infestation. Outbreaks in households and institutional settings further highlight the importance of coordinated treatment strategies to interrupt transmission (12).

From an economic perspective, scabies transmission generates a collective-action problem because individual treatment decisions create positive externalities that extend beyond the treated person. Effective control requires coordinated activity among household members and close contacts, but individuals may underinvest in treatment if they do not fully internalize the benefits conferred on others. This logic is closely related to the broader literature on collective action, public goods, and common-pool resource problems (16–18). The persistence of scabies despite the availability of effective treatments illustrates how individually rational behavior can produce socially inefficient outcomes, especially when treatment decisions are influenced by expectations about others' compliance.

The present framework also relates to the literature on coordination games, strategic complementarities, and health externalities. Treatment decisions depend on beliefs regarding whether others will also participate in control efforts, creating the possibility of multiple equilibria and coordination failure (19–21). Similar mechanisms have been documented in infectious-disease settings, where preventive actions generate spillover effects that individuals may fail to internalize (22–24). For example, Miguel and Kremer (23) found that that treating certain people reduced the risk of illness for others, resulting in positive externalities. This illustrates how individual rational decisions may not result in the socially optimal level of treatment. More broadly, recent evidence emphasizes that scabies transmission is influenced not only by biological factors but also by behavioral, environmental, and socioeconomic conditions, reinforcing the importance of coordinated responses and collective prevention strategies.

This paper argues that the persistence of scabies can be understood as a coordination failure problem. Using a formal game-theoretic framework, I demonstrate that rational individuals may converge to inefficient Nash equilibria characterized by incomplete treatment and ongoing transmission. This framework provides a unified explanation for delayed treatment, partial treatment, recurrent infection, and persistent disease burden despite the availability of therapies.

Beyond the static Nash equilibrium framework, this study extends the analysis with an evolutionary game-theoretic perspective based on replicator dynamics. This extension allows the examination of how treatment behaviors evolve over time in response to differences in expected payoffs and how populations may converge toward efficient or inefficient equilibria under alternative treatment-cost scenarios. In addition, the theoretical analysis is complemented by empirical evidence and robustness assessments based on multiple yearly specifications and regional comparisons of household washing machine coverage. Together, these analyses link the theoretical framework to observed patterns of scabies burden and provide additional insight into the factors associated with persistent transmission.

## 2 Theoretical framework

The objective of the theoretical framework is to develop a stylized game-theoretic representation of the coordination problem underlying scabies treatment decisions rather than a comprehensive epidemiological transmission model. The model is intentionally reduced-form and is designed to isolate the strategic interactions that arise when treatment decisions are interdependent. Treatment by one household member reduces the risk of infection for other household members, generating a positive externality analogous to herd protection. Conversely, an untreated household member may remain a source of infestation and reinfect a household member who has already received treatment. Heterogeneous contact networks and detailed transmission dynamics are abstracted in order to focus on the coordination incentives that motivate the subsequent evolutionary analysis.

### 2.1 The economic model

Consider a household in which each individual chooses one of two actions: *T* denotes receiving treatment, and *N* denotes not receiving treatment.

Let:

- *c* = private cost of treatment;
- *L* = full expected loss associated with infection;
- *p*(*k*) = residual probability that an untreated individual remains infected when the other household members are treated, conditional on the relevant pattern of household exposure *k*; and
- *r* = expected cumulative reinfestation loss incurred by a treated individual when the other household member remains untreated.

In the game-theoretic model, L represents the expected loss or damage from being infected with scabies. Biologically and clinically, scabies causes a range of pathological and social harms:

1. Skin damage and inflammation
  - Scabies mites burrow into the stratum corneum of the skin, causing intense pruritus (itching), erythema, and inflammation (25).
  - Lesions can appear as vesicles, papules, and crusts, especially in crusted scabies (26).
2. Secondary infections
  - Persistent scratching may break the skin barrier, leading to bacterial superinfections, commonly involving Staphylococcus aureus or Streptococcus pyogenes (27).
3. Systemic effects
  - In severe or untreated cases, especially in immunocompromised individuals, scabies can trigger widespread skin inflammation and contribute to secondary complications such as post-streptococcal glomerulonephritis (1).
4. Social and psychological impact
  - Scabies causes sleep disturbance, social stigma, and school/work absenteeism, which can be considered part of the loss in a game-theoretic sense (28).

The parameter *c* denotes the private treatment cost, whereas *L* denotes the full expected loss when neither household member treats. If one member treats, an untreated member faces the residual conditional infection probability *p*(*k*), where 0 < *p*(*k*) < 1, and therefore the reduced expected loss *p*(*k*)*L*.

The inequality *p*(*k*)*L*<*L* captures herd protection: treatment by one household member removes one potential source of transmission and reduces, without eliminating, the infection risk faced by the untreated member. The term “herd protection” is used because treatment reduces exposure but does not necessarily produce lasting biological immunity.

The untreated member is not fully protected because *p*(*k*) remains positive. The parameter *p*(*k*) is therefore defined directly as a conditional residual probability; no unused baseline transmission parameterization is imposed.

Conversely, an untreated household member may remain in a reservoir and reinfest a member who has completed treatment. The resulting loss *r* is cumulative: it encompasses not only reinfestation itself but also repeated medication, additional medical consultations, repeated laundering, productivity losses, persistent symptoms, and delayed household eradication.

The reinfestation loss and the residual infection probability describe related consequences of continued exposure and should not be treated as independent parameters. I therefore write

$$\begin{array}{l} r=\rho \left(p\left(k\right)\right),\;\rho^{\prime }\left(p\left(k\right)\right)>0. \end{array}$$

The function ρ(·) maps the conditional probability that the untreated household member remains infested into the cumulative expected loss imposed on the treated member. This loss may include repeated medication, additional consultations, repeated laundering, productivity losses, persistent symptoms, and delayed household eradication. No numerical functional form is imposed because these quantities are not estimated in the present study; monotonicity nevertheless prevents *r* from being selected independently of *p*(*k* ).

Under this link, the two conditions defining the coordination regime are

$$\begin{array}{l} c<p\left(k\right)L \end{array}$$

and

$$\begin{array}{l} c+\rho \left(p\left(k\right)\right)>L. \end{array}$$

Assuming that ρ(·) is strictly increasing, these conditions are equivalent to

$$\begin{array}{l} p\left(k\right)>\max\left\{\frac{c}{L},\rho^{-1}\left(L-c\right)\right\}. \end{array}$$

The coordination region is non-empty when

$$\begin{array}{l} \max\left\{\frac{c}{L},\rho^{-1}\left(L-c\right)\right\}<1. \end{array}$$

Thus, coordination is a conditional regime rather than an automatic feature of the model. A sufficient substantive interpretation is that *c*<*L* and that the expected cumulative reinfestation loss at sufficiently high conditional exposure risk can exceed *L*−*c*. Simultaneous-treatment recommendations are consistent with this possibility because untreated household contacts can remain continuing sources of reinfestation (14, 15).

The static model below is introduced to visualize the payoff ordering using evolutionary analysis. It is not intended as a complete epidemiological description of person-to-person transmission. The formal analysis of treatment adoption, equilibrium stability, and long-run behavior is developed in Section 2.3 through replicator dynamics.

The reduced-form utility functions are:

$$\begin{array}{l} U\left(T,T\right)=-c \end{array}$$ (1)

$$\begin{array}{l} U\left(T,N\right)=-\left(c+r\right) \end{array}$$ (2)

$$\begin{array}{l} U\left(N,T\right)=-p\left(k\right)L \end{array}$$ (3)

$$\begin{array}{l} U\left(N,N\right)=-L \end{array}$$ (4)

Equation 1 represents coordinated treatment: when both household members treat, each incurs only the treatment cost c. Equation 2 represents unilateral treatment: the treated individual incurs the treatment cost and remains exposed to the expected reinfection loss *r* because the other household member does not treat. Equation 3 represents the herd-protection benefit an untreated individual receives when the other household member is treated. Equation 4 represents the full expected loss when neither household member treats.

When the other household member treats, the individual compares *U*(*T, T*) = −*c with U*(*N, T*) = −*p*(*k*)*L*. Treatment is preferred when

$$\begin{array}{l} -c>-p\left(k\right)L,\text{or}\;\text{equivalently},c<p\left(k\right)L \end{array}$$ (5)

When the other household member does not treat, the individual compares *U*(*T, N*) = −(*c* + *r*) with *U*(*N, N*) = −*L*. Non-treatment is preferred when

$$\begin{array}{l} -L>-\left(c+r\right),\text{or}\;\text{equivalently},c+r>L \end{array}$$ (6)

Taken together, Equations 5 and 6 imply the payoff ordering −*c* > −*p*(*k*)*L* > −*L* > −(*c* + *r*). The first inequality makes treatment the best response to treatment; the middle inequality represents herd protection; and the final inequality makes non-treatment the best response to non-treatment. The second condition is substantive rather than automatic and, under *r* = ρ(*p*(*k*)), cannot be obtained by selecting *r* independently of exposure risk.

Within this parameter region, neither strategy is dominant. Treatment is the preferred response to treatment, whereas non-treatment is the preferred response to non-treatment. The static game therefore has two pure-strategy Nash equilibria: (T,T) and (N,N). Other parameter regions generate free-riding or dominant strategies and are mapped in Section 2.3.

The static representation is deliberately parsimonious. Its purpose is to provide a transparent visual bridge to the population-level expected payoffs in Equations 7, 8, rather than to serve as a separate epidemiological model.

### 2.2 Nash equilibrium analysis

Under the coordination restrictions *c*<*p*(*k*)*L* and *c* + *r* > *L*, the best-response correspondence yields two pure-strategy Nash equilibria.

First, (*T, T*) is a Nash equilibrium because, when the other household member treats, deviating from treatment to non-treatment changes the payoff from −*c* to −*p*(*k*)*L*. Since *c*<*p*(*k*)*L*, it follows that −*c* > −*p*(*k*)*L*, and treatment is preferred. This equilibrium reflects successful coordination: both members incur the treatment cost, the within-household reservoir is removed, and neither remains exposed to reinfestation by the other.

Second, (*N, N*) is a Nash equilibrium because, when the other household member does not treat, deviating from non-treatment to treatment changes the payoff from −*L* to −(*c* + *r*). Since *c* + *r* > *L*, it follows that −*L* > −(*c* + *r*), and non-treatment is preferred. This equilibrium reflects coordination failure: although joint treatment would remove the within-household source of reinfestation, neither individual wishes to treat unilaterally.

The two equilibrium exist only in the coordination regime. Expectations then determine which equilibrium is reached, and the unstable interior threshold separating their basins of attraction is derived from Section 2.3. The model does not assume that bistability applies for every possible parameter configuration.

#### 2.2.1 2 × 2 game illustration

Table 1 presents the reduced-form static game between two representative household members. Each player chooses Treat (T) or Not Treat (N). The matrix is symmetric: the off-diagonal cells are transposed to one another, and both players are governed by the same payoff function.

**Table 1:** Payoff matrix for household treatment decisions.

	Neighbor treats (T)	Neighbor does not treat (N)
You treat (T)	(−*c*, −*c*)	(−(*c* + *r*), −*p*(*k*)*L*)
You do not treat (N)	(−*p*(*k*)*L*, −(*c* + *r*))	(−*L*, −*L*)

*c* denotes the treatment cost, *L* the full expected infection loss, and *p*(*k*) the residual conditional probability of infection faced by an untreated individual when the other member is treated. The expected cumulative reinfestation loss is *r* = ρ(*p*(*k*)), with ρ′(*p*(*k*)) > 0; *r* is written separately in the matrix for readability. The first entry in each cell is the row player's payoff and the second is the column player's payoff.

The table presents the stylized conceptual model in two-player form. Its role is to display the payoff ordering used to construct evolutionary expected payoffs. The formal model of population behavior begins in Section 2.3.

For a player choosing treatment, the payoff is −*c* when the other household member also treats and −(*c* + *r*) when the other household member does not treat. For a player choosing non-treatment, the payoff is −*p*(*k*)*L* when the other household member treats and –*L* when the other household member also does not treat.

The population-level expected payoff from treatment is obtained by averaging the two treatment payoffs in Table 1. A person choosing treatment encounters another treated individual with probability x and an untreated individual with probability 1– x. Therefore,

$$\begin{array}{l} f_{T}\left(x\right)=x\left(-c\right)+\left(1-x\right)\left[-\left(c+r\right)\right]=-c-\left(1-x\right)r \end{array}$$ (7)

The first derivative of Equation 7 with respect to the treatment share *x* is:

$$\begin{array}{l} \frac{df_{T}\left(x\right)}{dx}=r>0. \end{array}$$

Thus, the expected payoff from treatment increases as the proportion of treated individuals rises. The reason is that a higher treatment share reduces the probability 1−*x* that a treated individual encounters an untreated source of infestation. Consequently, the expected reinfection loss declines as treatment coverage increases. This result is consistent with the epidemiological logic of scabies control: coordinated treatment lowers the risk that successfully treated individuals will be reinfected by untreated household or community members.

Equation 7 shows that the expected payoff from treatment consists of the direct treatment cost *c* and the expected reinfection loss. The reinfection loss is weighted by 1 – x, the probability of encountering an untreated individual. As the treatment share x increases, the probability of exposure to an untreated source of infestation declines, and the expected loss of reinfection becomes smaller. At x = 1, coordinated treatment is universal, the reinfection term disappears, and the treatment payoff is −*c*. At *x* = 0, a treated individual faces the maximum expected reinfection loss, and the payoff is −(*c* + *r*).

The population-level expected payoff from non-treatment is obtained by averaging the two non-treatment payoffs in Table 1. A person choosing non-treatment encounters a treated individual with probability x and an untreated individual with probability 1 − *x*. Therefore,

$$\begin{array}{ll} f_{N}(x) & =x[-p(k)L]+(1-x)(-L)=-[xp(k)L\\ & +(1-x)L] \end{array}$$ (8)

The first derivative of Equation 8 with respect to the treatment share is

$$\begin{array}{l} \frac{df_{N}\left(x\right)}{dx}=L\left[1-p\left(k\right)\right]>0, \end{array}$$

Equation 8 incorporates the herd-protection effect. When an untreated individual encounters a treated individual, which occurs with probability x, the expected loss is reduced from *L* to *p*(*k*)*L*. When the untreated individual encounters another untreated individual, which occurs with probability 1 − *x*, the full expected loss *L* applies. As x increases, greater weight is placed on the reduced loss *p*(*k*)*L*, and the expected payoff from non-treatment improves. Thus, untreated individuals can benefit from the lower transmission risk created by treatment adoption among others.

At *x* = 0, no other individual treats, and Equation 8 gives *f*_*N*_(0) = −*L*. At *x* = 1, all other individuals treat, and Equation 8 gives *f*_*N*_(1) = −*p*(*k*)*L*. The difference between −*L* and −*p*(*k*)*L* represents the herd-protection benefit an untreated individual receives as treatment adoption increases. Because *p*(*k*) remains positive; the untreated individual is not fully protected even when all others are treated.

Equations 7, 8 therefore follow directly from the four payoffs presented in Table 1. Equation 7 captures the reinfection externality imposed on treated individuals by untreated household members, whereas Equation 8 captures the herd-protection externality received by untreated individuals from treated household members. Together, these opposing externalities generate the strategic interdependence underlying the coordination problem.

Under *c*<*p*(*k*)*L* and *c* + *r* > *L*, treatment is optimal against treatment, and non-treatment is optimal against non-treatment. The game then admits the two pure-strategy Nash equilibria (*T, T*) and (*N, N*). Section 2.3 shows how alternative signs at these two boundaries generate the other parameter regimes.

Because the table is used to visualize the reduced-form strategic structure, the subsequent replicator dynamics provide a formal analysis of how treatment strategies evolve across the population.

### 2.3 Evolutionary dynamics framework

The evolutionary framework models treatment and non-treatment as strategies whose population frequencies change according to their expected payoffs. The two-player household game provides the micro-foundation, while the replicator dynamics offer a stylized representation of how treatment strategies may spread across a population of households (35). They are not intended to describe behavioral adaptation within a single household.

Let *x*∈[0, 1] denote the fraction of the population choosing treatment, so that 1−*x* denotes the fraction choosing non-treatment. The parameters *c*, *L*, and *p*(*k*) retain their definitions from Section 2.1, and *r* = ρ(*p*(*k*)) is the expected reinfestation loss associated with encountering an untreated individual. Throughout the analysis, *r* is used as shorthand for this increasing function of the conditional probability of infection; it is not an independently chosen parameter.

**a) Payoff functions**

The expected payoff from treatment is

$$\begin{array}{l} f_{T}\left(x\right)=x\left(-c\right)+\left(1-x\right)\left[-\left(c+r\right)\right]=-c-\left(1-x\right)r. \end{array}$$ (7)

The expected payoff from non-treatment is

$$\begin{array}{l} f_{N}\left(x\right)=x\left[-p\left(k\right)L\right]+\left(1-x\right)\left(-L\right). \end{array}$$ (8)

The average payoff in the population is

$$\begin{array}{l} \bar{f}\left(x\right)=xf_{T}\left(x\right)+\left(1-x\right)f_{N}\left(x\right). \end{array}$$ (9)

Substitution of Equations 7 and 8 gives

$$\begin{array}{ll} \bar{f}\left(x\right) & =x\left[-c-\left(1-x\right)r\right]+\left(1-x\right)\;-xp\left(k\right)L\\ & -\left(1-x\right)L. \end{array}$$ (10)

**b) Replicator equation**

The treatment share evolves according to

$$\begin{array}{l} ẋ=x\left[f_{T}\left(x\right)-\bar{f}\left(x\right)\right]. \end{array}$$ (11)

Equations 9–11 define the average payoff and the corresponding replicator dynamics. With two strategies, this expression can be written as ẋ = *x*(1−*x*)[*f*_*T*_(*x*)−*f*_*N*_(*x*)]. Substituting Equations 7, 8 and collecting terms yields

$$\begin{array}{l} ẋ=x\left(1-x\right)\left\{L-c-r+x\left[r-L\left(1-p\left(k\right)\right)\right]\right\}. \end{array}$$ (12)

The derivation is reported in Appendix A1. Define the payoff advantage of treatment as

$$\begin{array}{l} \Delta \left(x\right)=f_{T}\left(x\right)-f_{N}\left(x\right)=L-c-r+x\left[r-L\left(1-p\left(k\right)\right)\right]. \end{array}$$

At the two boundaries,

$$\begin{array}{l} \Delta \left(0\right)=L-c-r \end{array}$$

and

$$\begin{array}{l} \Delta \left(1\right)=p\left(k\right)L-c. \end{array}$$

These two expressions determine the type of game and the stability of the boundary equilibria. Treatment increases when Δ(*x*)>0 and decreases when Δ(*x*) < 0.

**c) Equilibria**

Setting Equation 12 equal to zero gives

$$\begin{array}{l} x\left(1-x\right)\left\{L-c-r+x\left[r-L\left(1-p\left(k\right)\right)\right]\right\}=0. \end{array}$$ (13)

The boundary equilibria are *x* = 0 and *x* = 1. When the denominator is non-zero, an interior equilibrium is

$$\begin{array}{l} x^{*}=\frac{c+r-L}{r-L\left[1-p\left(k\right)\right]}. \end{array}$$ (14)

Equations 13 and 14 identify the boundary and interior equilibria of the model. Whether x^*^ lies in the unit interval and whether it is stable depend on the signs of Δ(0) and Δ(1), as shown below and derived formally from Appendix A2.

**d) Parameter regimes**

The model generates four logically possible regimes. Three are central to the present analysis; the fourth is stated for completeness.

Coordination and bistability arise when

$$\begin{array}{l} c+r>L\;\text{and}\;c<p\left(k\right)L. \end{array}$$

Treatment is then disadvantageous when adoption is near zero but advantageous when adoption is near one. Both boundary equilibrium are locally stable, while the interior equilibrium satisfies 0 < x^*^ < 1 and is unstable. It therefore acts as a threshold: initial adoption below x^*^ converges toward no treatment, whereas adoption above x^*^ converges toward full treatment.

Free-riding with a stable interior equilibrium arises when

$$\begin{array}{l} c+r<L\;\text{and}\;c>p\left(k\right)L. \end{array}$$

Treatment is attractive when few others treat, but non-treatment becomes attractive at high coverage because untreated individuals benefit from herd protection. Both boundary equilibria are unstable, and the interior equilibrium is locally stable.

Treatment is strictly dominant when

$$\begin{array}{l} c+r<L\;\text{and}\;c<p\left(k\right)L, \end{array}$$

or equivalently *c*<*min*{*L*−*r, p*(*k*)*L*}. The payoff advantage of this treatment is positive at both boundaries and therefore throughout the unit interval. The interior threshold disappears, *x* = 0 is unstable, and *x* = 1 is locally stable. A sufficiently large reduction in treatment cost can therefore eliminate the coordination problem rather than merely moving its threshold.

For completeness, non-treatment is strictly dominant when *c*+*r*>*L* and *c*>*p*(*k*)*L*, or *c*>*max*{*L*−*r, p*(*k*)*L*}. In that case, *x* = 0 is locally stable and *x* = 1 is unstable.

Because *r* = ρ(*p*(*k*)), the coordination regime may also be written as

$$\begin{array}{l} p\left(k\right)>\max\left\{\frac{c}{L},\rho^{-1}\left(L-c\right)\right\}. \end{array}$$

It is non-empty when

$$\begin{array}{l} \max\left\{\frac{c}{L},\rho^{-1}\left(L-c\right)\right\}<1. \end{array}$$

A sufficient substantive interpretation is that *c*<*L* and that, at sufficiently high conditional exposure risk, the cumulative expected reinfestation loss can exceed *L*−*c*. These restrictions make clear that coordination is conditional on the underlying epidemiological and economic environment; it is not imposed by selecting *r* independently of *p*(*k* ).

### 2.4 Discussion of the theoretical model results

The model does not imply that scabies treatment always constitutes a coordination game. Coordination and bistability arise only when joint treatment is individually preferable to free-riding, *c*<*p*(*k*)*L*, while unilateral treatment is less attractive than mutual non-treatment, *c*+*r*>*L*. In this region, expectations matter because treatment is advantageous only after adoption exceeds the unstable interior threshold.

The condition *c*+*r*>*L* is substantive. It states that when another household member remains untreated, the combined cost of treatment and the expected cumulative consequences of reinfestation exceed the loss associated with remaining untreated. The term *r* includes not only the biological event of reinfestation but also repeated medication, additional medical consultations, repeated laundering, productivity losses, persistent symptoms, and delayed household eradication. Clinical recommendations that all household members and close contacts be treated simultaneously reflect the importance of these cumulative consequences (14, 15).

Policies may change the type of game. In particular, reducing *c* below both *L*−*r* and *p*(*k*)*L* makes treatment strictly dominant. The intervention then removes the unstable threshold instead of merely lowering it. Conversely, when herd protection makes non-treatment attractive at high coverage while treatment remains attractive at low coverage, the model generates a stable interior free-riding equilibrium.

The principal theoretical conclusion is therefore conditional: reinfestation externalities can generate a coordination trap, but sufficiently strong cost reductions or coordinated delivery mechanisms can eliminate that trap. This distinction sharpens the policy rationale for subsidies, simultaneous household treatment, and mass drug administration.

### 2.5 Limitations of the theoretical framework

The framework is intentionally reduced-form and should be interpreted as a conceptual representation of strategic treatment interdependence rather than as a calibrated epidemiological transmission model. The model links reinfestation loss monotonically to conditional infection risk through *r* = ρ(*p*(*k*)), but it does not impose a specific functional form because these parameters are not estimated in the present study.

The analysis also assumes homogeneous individuals and abstracts from variation in treatment effectiveness, adherence, household size, contact networks, age structure, asymptomatic infestation, access to healthcare, and repeated transmission over time. These factors could alter both the location of the regime boundaries and the dynamics of treatment adoption.

Finally, replicator dynamics describe the evolution of strategy frequencies across a population of households, not learning within a particular household. Future research could embed the strategic mechanism in a richer epidemiological model and estimate the function ρ(·) using household-level longitudinal data.

Despite these limitations, the model identifies transparent conditions under which reinfestation externalities produce coordination, free-riding, or dominant-strategy outcomes and clarifies how policy can move the system between these regimes.

## 3 Empirical illustration, public policy, and urban implementation

Empirical evidence underscores the substantial global burden of scabies, particularly in resource-limited and overcrowded settings (1, 29, 30). Fiji provides a notable example, where prevalence has reached approximately 30% of the population. Studies further demonstrate that the Mass Drug Administration (MDA) can reduce prevalence by more than 70%, but without sustained coordination and follow-up interventions, infection rates may rebound over time, highlighting the importance of continuous control efforts (11). These findings illustrate both the potential and the limitations of large-scale control programs and motivate the discussion below.

### 3.1 Empirical illustration

The experience of Fiji illustrates both the effectiveness and the limitations of large-scale scabies control programs. Scabies cases are defined according to the international consensus diagnostic criteria developed by Engelman et al. (31). Population-based studies report prevalence rates ranging from 0.18% to 76.9%, with the highest rates observed in tropical and low-income regions (7). Global estimates suggest that hundreds of millions of individuals are affected annually, with repeated outbreaks exacerbated by overcrowding and inadequate hygiene infrastructure (15). The disease causes intense pruritus, sleep disruption, secondary bacterial infections, and social stigma, collectively contributing to substantial morbidity and economic burden (6, 32).

Fiji provides a particularly informative example of these challenges. In some communities, scabies prevalence has reached approximately 30% of the population. Although MDA programs have reduced prevalence by more than 70%, infection rates may rebound when coordinated treatment efforts are not maintained, underscoring the importance of sustained surveillance, community engagement, and follow-up interventions.

The theoretical analysis implies that persistent low-treatment outcomes depend on the relationship among treatment cost, infection loss, and reinfestation loss rather than on an assumed numerical calibration. Public policy can alter these relationships through subsidies, free topical treatments, or mass drug administration campaigns, thereby promoting the socially optimal full-treatment outcome. Beyond pharmacological interventions, hygiene practices are critical: washing bedding, clothing, and linens at temperatures of at least 60 °C can markedly reduce reinfection. However, in many low-income or rural areas, including parts of Sub-Saharan Africa and India, laundering is done by hand in rivers or other communal water sources, often limiting the effectiveness of this preventive measure.

An urban economics approach can help overcome these structural barriers. Establishing communal washing centers equipped with high-temperature washing machines can reduce logistical constraints and infection risks, particularly in dense urban settlements. These centers not only provide accessible hygiene infrastructure but can also serve as focal points for education and community engagement, reinforcing coordinated treatment behavior. Integrating such infrastructure with MDA programs and targeted health education campaigns aligns individual incentives with social welfare and reduces the likelihood of returning to inefficient, low-treatment equilibria. This evidence suggests that sustainable scabies control requires a multifaceted policy strategy that combines medical, behavioral, and infrastructural interventions.

### 3.2 Public policy implications

The persistence of scabies, as demonstrated in the theoretical framework above, reflects a classic case of coordination failure under positive externalities. These theoretical results have direct implications for public policy.

According to the theoretical model, a coordination trap arises when treatment is unattractive at low adoption even though universal treatment is stable. In that parameter regime, expectations of limited participation can trap populations in a low-treatment equilibrium and sustain scabies transmission.

Public policy can reduce the effective treatment cost through subsidies, free distribution of topical medications, or mass drug administration. Within the coordination regime, reducing treatment cost lowers the unstable adoption threshold; a sufficiently large reduction changes the game more fundamentally by making treatment strictly dominant. The policy objective is therefore not merely to shift behavior within a fixed game, but potentially to eliminate the coordination problem itself. Context-specific hygiene infrastructure and community education can complement this change by reducing the risk of reinfestation and supporting simultaneous treatment.

#### 3.2.1 Subsidization and financial incentives

One of the most direct policy tools is the subsidization of treatment. By reducing the private treatment cost *c*, subsidies increase the likelihood that the individual treatment condition derived in Section 2.1,

$$\begin{array}{l} c\leq p\left(k\right)L, \end{array}$$

is satisfied. Consequently, treatment becomes individually rational for a larger share of the population. Because treatment decisions exhibit strategic complementarities, this increase in individual incentives can shift the population toward the socially optimal full-treatment equilibrium.

In practical terms, subsidies lower the effective treatment cost *c*, making treatment attractive to more individuals. As treatment adoption increases, coordinated participation becomes more likely, reducing persistent transmission and improving collective welfare.

Empirical evidence from neglected tropical disease (NTD) programs suggests that even small reductions in cost can significantly increase treatment uptake. Importantly, because treatment decisions exhibit strategic complementarities, subsidies can generate multiplier effects: as more individuals choose treatment, the marginal benefit of treatment increases for others, potentially tipping the system toward a full-treatment equilibrium.

However, subsidies alone may be insufficient in high-prevalence settings where expectations of non-compliance remain entrenched.

#### 3.2.2 Mass drug administration (MDA) as a coordination mechanism

Mass drug administration represents a powerful intervention that directly addresses coordination failures. By providing treatment to all individuals within a target population simultaneously, MDA effectively eliminates strategic uncertainty.

In game-theoretic terms, MDA collapses the strategy space:

$$\begin{array}{l} a_{i}=T\forall i \end{array}$$

The expression indicates that all individuals receive treatment simultaneously. This removes uncertainty about whether others will comply, thereby eliminating the coordination problem that sustains transmission. As a result, MDA can move the population directly to the socially optimal outcome in which reinfection risk is minimized.

Evidence from Fiji demonstrates that community-wide MDA can reduce scabies prevalence by more than 70% within a relatively short time frame (11). Nevertheless, sustainability remains a key challenge. Without repeated rounds of MDA or complementary behavioral interventions, populations may revert to inefficient equilibria due to the reintroduction of the disease and declining compliance.

Thus, MDA should be understood not as a one-time solution but as part of a broader dynamic strategy.

#### 3.2.3 Information, beliefs, and health education

Information asymmetry and incorrect beliefs play a central role in sustaining the inefficient equilibria. Individuals may underestimate the contagiousness of scabies, the necessity of simultaneous treatment, or the long-term costs of reinfection.

Health education campaigns can shift beliefs along three key dimensions:

1. Perceived infection risk
2. Expected behavior from others
3. Perceived effectiveness of treatment

From a game-theoretic perspective, these interventions influence equilibrium selection. When individuals believe that others are likely to treat, their own incentive to treat increases, making high-treatment equilibria self-reinforcing.

Behavioral interventions—such as community engagement, peer education, and culturally adapted messaging—have been shown to significantly improve compliance in NTD programs.

#### 3.2.4 Institutional policies and regulation

In institutional environments—such as schools, prisons, and long-term care facilities—centralized policies can effectively eliminate coordination problems.

Such policies may include:

- Mandatory screening
- Isolation protocols
- Simultaneous treatment mandates

Because these environments allow for monitoring and enforcement, they are particularly well-suited to overcome coordination failures.

Outbreaks in high-income countries often originate in such settings, highlighting the importance of targeted institutional policies even in otherwise low-prevalence populations.

#### 3.2.5 Integrated policy design

The analysis suggests that no single intervention is sufficient. Instead, effective policy requires a multi-layered approach that simultaneously addresses costs, coordination, and information.

An optimal policy mix includes:

- Subsidies to reduce financial barriers
- MDA to achieve rapid prevalence reduction
- Structural hygiene interventions, including investments in household and community hygiene infrastructure (e.g., improved access to washing facilities and high-temperature laundering), complement medical interventions and reduce transmission risk.
- Education to sustain behavioral change
- Institutional regulation to prevent localized outbreaks

This integrated framework aligns private incentives with social welfare and reduces the likelihood of reverting to inefficient equilibria.

#### 3.2.6 Dynamic considerations and policy persistence

A critical implication of the model is path dependence. Once a population has converged on a low-adoption equilibrium, it may remain stable even with reduced intervention. Conversely, populations trapped in low-treatment equilibria may require substantial initial intervention to shift their trajectories.

This suggests that:

- Early intervention yields high returns
- Temporary policies can have long-term effects if they shift expectations
- Monitoring and follow-up are essential to prevent relapse

## 4 Exploratory empirical analysis

The empirical component provides an illustrative country-level assessment of the relationship between household hygiene infrastructure and scabies burden. The unit of observation is the country. The dependent variable is the natural logarithm of the 2021 age-standardized scabies disability-adjusted life-year (DALY) rate per 100,000 population for both sexes. The principal explanatory variable is the percentage of households with a washing machine, measured separately in 2018, 2019, 2020, and 2021. Because the analysis is observational and cross-sectional, the estimated coefficients are interpreted as descriptive conditional associations rather than causal effects.

The DALY data were obtained from the Global Burden of Disease Study 2021 dataset reported by Li et al. (5, 34), from which the 2021 age-standardized scabies DALY rate was extracted. Data on household washing machine coverage were obtained from the Global Data Lab (36). The original Global Data Lab worksheet contained both national and subnational observations. To ensure that each country contributed no more than one observation, I retained only records identified as “National” and “Total.” The resulting national observations were then merged one-to-one with the country-level DALY data. Sample sizes vary across specifications because washing machine coverage is not available in every country in every year.

Separate ordinary least squares regressions were estimated using washing machine coverage from each of the four measurement years (33). In the global sample, all specifications include continent indicators to account for broad regional differences, and heteroskedasticity-robust *p-values* are reported. A second set of regressions is restricted to countries classified by the Global Data Lab as Asia/Pacific. The four annual specifications are intended as a timing-sensitivity analysis: they examine whether the estimated association depends materially on the year in which washing machine coverage is measured. They should not be interpreted as four independent corroborating tests.

Table 2 reports the results for the global sample with continent controls. The estimated coefficient on washing machine coverage is negative in every specification, ranging from −0.0060 in 2018 to −0.0074 in 2021, and is statistically significant at the 10% level in all four regressions. Because the dependent variable is logarithmic, the estimates imply that a one-percentage-point increase in household washing machine coverage is associated with an approximately 0.6%−0.7% lower scabies DALY rate, conditional on the included continent indicators. The number of countries ranges from 50 to 63, and the R-squared values range from 0.394 to 0.445. These findings indicate a stable negative conditional association, but they do not establish a causal effect of washing machine ownership on scabies burden.

**Table 2:** Relationship between scabies DALYs and household washing machine coverage: global sample with continent controls (2018–2021).

Variables	(1) ln(DALY)	(2) ln(DALY)	(3) ln(DALY)	(4) ln(DALY)
W2018	−0.0060^*^	–	–	–
	(0.0882)	–	–	–
W2019	–	−0.0067^*^	–	–
	–	(0.0788)	–	–
W2020	–	–	−0.0070^*^	–
	–	–	(0.0710)	–
W2021	–	–	–	−0.0074^*^
	–	–	–	(0.0690)
Continent controls	Yes	Yes	Yes	Yes
Observations	63	58	55	50
R-squared	0.399	0.400	0.394	0.445
Calculated F-value	38.64^***^	40.29^***^	33.96^***^	29.31^***^
d.f. numerator	4	4	4	4
d.f. denominator	58	53	50	45
1% critical F-value	3.66	3.70	3.72	3.77

• The dependent variable is the natural logarithm of the 2021 age-standardized scabies DALY rate per 100,000 population for both sexes.

• W2018–W2021 denote the percentage of households with a washing machine in each country, measured in the corresponding year.

• Each column reports a separate country-level timing-sensitivity regression; the DALY outcome is measured in 2021 in all columns.

• All specifications include continent indicators. Their coefficients are omitted for brevity.

• Because the dependent variable is logarithmic, 100[exp(β)−1] gives the approximate percentage difference in the DALY rate associated with a one-percentage-point increase in washing machine coverage.

• Heteroskedasticity-robust p-values are reported in parentheses. Significance levels: ^***^p < 0.01, ^*^p < 0.10.

• Negative coefficients indicate a conditional association between greater washing machine coverage and lower scabies DALY rates. The estimates are exploratory and should not be interpreted as causal.

### 4.1 Robustness and interpretation

Country-level DALY estimates were available only for 2021. Accordingly, the outcome is held constant while washing machine coverage is measured from 2018 to 2021. Household appliance ownership generally changes gradually, making earlier measurements potentially informative about the prevailing household infrastructure. The similarity in the signs and magnitudes of the coefficients across the four specifications indicates that the estimated association is not highly sensitive to the measurement year. Nevertheless, the strong persistence of washing machine coverage over time means that these regressions are closely related specifications rather than independent replications.

Table 3 presents the exploratory analysis for the Asia/Pacific sample. The estimated coefficient on washing machine coverage remains negative across all four specifications, ranging from −0.0115 to −0.0139. Each coefficient is statistically significant at the 5% level. In log-linear terms, a one-percentage-point increase in washing machine coverage is associated with an approximately 1.1%−1.4% lower scabies DALY rate in this subsample. The estimates are somewhat larger in absolute value than those obtained from the global sample, although the Asia/Pacific regressions contain only 22–27 countries and do not include additional covariates. The regional results should therefore be regarded as exploratory evidence of heterogeneity, not as an independent causal test or as proof that washing machine ownership itself reduces the scabies burden.

**Table 3:** Relationship between scabies DALYs and household washing machine coverage: Asia/Pacific sample (2018–2021).

Variables	(1) ln(DALY)	(2) ln(DALY)	(3) ln(DALY)	(4) ln(DALY)
Constant	4.7351^***^	4.7878^***^	4.8186^***^	4.9438^***^
	(< 0.01)	(< 0.01)	(< 0.01)	(< 0.01)
W2018	−0.0115^**^	–	–	–
	(0.0236)	–	–	–
W2019	–	−0.0125^**^	–	–
	–	(0.0183)	–	–
W2020	–	–	−0.0128^**^	–
	–	–	(0.0153)	–
W2021	–	–	–	−0.0139^**^
	–	–	–	(0.0135)
Observations	27	25	25	22
R-squared	0.131	0.149	0.157	0.197
Calculated F-value	5.81^**^	6.45^**^	6.86^**^	7.33^**^
d.f. numerator	1	1	1	1
d.f. denominator	25	23	23	20
1% critical F-value	7.77	7.88	7.88	8.10

• The dependent variable is the natural logarithm of the 2021 age-standardized scabies DALY rate per 100,000 population for both sexes.

• The sample is restricted to countries classified as Asia/Pacific by Global Data Lab. Only national observations identified as “National” and “Total” are retained.

• W2018–W2021 denote the percentage of households with a washing machine in each country, measured in the corresponding year.

• Each column reports a separate country-level timing-sensitivity regression; the DALY outcome is measured in 2021 in all columns.

• Because the dependent variable is logarithmic, 100[exp(β)−1] gives the approximate percentage difference in the DALY rate associated with a one-percentage-point increase in washing machine coverage.

• Heteroskedasticity-robust p-values are reported in parentheses. Significance levels: ^***^p < 0.01, ^**^p < 0.05.

• Negative coefficients indicate that greater washing machine coverage is associated with lower scabies DALY rates in the Asia/Pacific sample. The estimates are exploratory and should not be interpreted as causal.

### 4.2 Dataset limitations

The empirical illustration is subject to several important limitations. First, both datasets are aggregated at the country level and cannot capture within-country variation in household conditions, scabies exposure, treatment behavior, or disease burden. Country-level associations must therefore not be interpreted as relationships at the household or individual level. Second, washing machine coverage may serve as a proxy for broader socioeconomic development, including income, housing quality, access to healthcare, sanitation, education, and institutional capacity. Although the global regressions include continent indicators, the analysis does not control comprehensively for these potential confounders. Third, the outcome is observed only for 2021, preventing a longitudinal assessment of changes in scabies burden. Fourth, incomplete availability of national washing machine data produces relatively small and changes samples across specifications, particularly in the Asia/Pacific analysis. Finally, the observational cross-sectional design does not permit causal identification. The empirical results should consequently be viewed as exploratory evidence consistent with the theoretical emphasis on household infrastructure, rather than as a definitive test of the proposed mechanism or a basis for standalone policy conclusions.

## 5 Conclusion

This study provides a comprehensive explanation for why scabies persists despite the availability of effective treatments. Theoretical modeling demonstrates that household treatment decisions constitute a coordination game in which individually rational behavior can lead to inefficient equilibria, sustaining infection cycles. To complement the theoretical analysis, the study presents an illustrative cross-country empirical analysis. The empirical results are consistent with the proposed theoretical framework: structural factors such as overcrowding and limited hygiene infrastructure are associated with persistent scabies burden, while greater household washing machine coverage is negatively associated with scabies DALYs. However, these findings should be interpreted as correlational rather than causal, since the empirical analysis does not control for broader socioeconomic and institutional determinants of scabies burden.

The analysis reveals a divergence between private and social incentives: individual treatment costs are low, yet societal costs—including reinfection, healthcare utilization, and productivity loss—are substantial. Public policy interventions, such as subsidized treatments, mass drug administration, health education, and improved urban hygiene infrastructure, can shift populations toward socially optimal equilibria by aligning individual behavior with collective welfare.

Ultimately, scabies should be viewed not only as a medical condition but also as a coordination problem. The principal contribution of this study is the development of a game-theoretic framework for understanding persistent scabies transmission, while the empirical analysis serves as an illustrative complement that is consistent with the proposed mechanism. Sustainable control requires integrating epidemiological insights, behavioral economics, and infrastructure-based interventions, with early, coordinated, and context-sensitive policies being essential to break persistent transmission cycles and reduce the global burden of disease.

## Data Availability

The datasets presented in this study can be found in online repositories. The names of the repository/repositories and accession number(s) can be found in the article/Supplementary material.
