# Supplementary material for: Why does scabies persist despite effective treatment? Coordination failure, Nash equilibrium, and replicator dynamics
Source: Front Public Health. 2026 Sep 16;14:1865428. doi: 10.3389/fpubh.2026.1865428 (PMC13623885; doi:10.3389/fpubh.2026.1865428)
Supplement: Supplementary file 1 [file Supplementary_File_1.pdf]

## Appendix A. Derivation of the Replicator Dynamics Equation

### Appendix A1. Substitution into the Replicator Equation

The expected payoff from treatment is given by Equation (7):

$$f_T(x) = x(-c) + (1-x)\big(-(c+r)\big).$$

Rearranging terms gives

$$f_T(x) = -c - r + rx.$$

The expected payoff from non-treatment is given by Equation (8):

$$f_N(x) = x(-p(k)L) + (1-x)(-L).$$

Rearranging terms gives

$$f_N(x) = -L + xL[1 - p(k)].$$

The average payoff in the population is

$$\bar{f}(x) = xf_T(x) + (1-x)f_N(x).$$

The replicator equation is

$$\dot{x} = x\big[f_T(x) - \bar{f}(x)\big].$$

Substituting the expression for the average payoff yields

$$\dot{x} = x\Big[f_T(x) - \big(xf_T(x) + (1-x)f_N(x)\big)\Big].$$

Since there are only two strategies,

$$\dot{x} = x(1-x)[f_T(x) - f_N(x)].$$

Substituting the expected payoff functions gives

$$\dot{x} = x(1-x)[(-c - r + rx) - (-L + xL[1 - p(k)])].$$

Collecting constant and linear terms,

$$\dot{x} = x(1-x)[L - c - r + x(r - L[1 - p(k)])].$$

Therefore, the evolutionary dynamics are

$$\boxed{\dot{x} = x(1-x)[L - c - r + x(r - L[1 - p(k)])],}$$

which is Equation (12) in the main text.

The first factor,

$$x(1-x),$$

ensures that the two boundary states,

$$x = 0 \text{ and } x = 1,$$

are always stationary. The term inside the square brackets represents the payoff advantage of treatment relative to non-treatment and therefore determines the direction of the evolutionary dynamics throughout the interior of the state space.

**Appendix A2. Jacobian Analysis of the Replicator Dynamics**

The replicator dynamics derived in Equation (12) is

$$\dot{x} = x(1-x)\left[L - c - r + x\left(r - L\big(1 - p(k)\big)\right)\right].$$

To simplify the notation, define

$$F(x) = x(1-x)$$

and

$$G(x) = L - c - r + x\left(r - L\big(1 - p(k)\big)\right).$$

The replicator equation can therefore be written as

$$\dot{x} = F(x)G(x).$$

The Jacobian of this one-dimensional dynamic system is simply the derivative of the right-hand side with respect to $x$:

$$J(x) = \frac{d\dot{x}}{dx}.$$

Applying the product rule,

$$J(x) = F'(x)G(x) + F(x)G'(x).$$

The derivative of the first term is

$$F'(x) = 1 - 2x,$$

while

$$G(x) = L - c - r + x\left(r - L\big(1 - p(k)\big)\right),$$

which implies

$$G'(x) = r - L\big(1 - p(k)\big).$$

Therefore,

$$\boxed{J(x) = (1 - 2x)\left[L - c - r + x\left(r - L\big(1 - p(k)\big)\right)\right] + x(1 - x)\big[r - L\big(1 - p(k)\big)\big].}$$

This expression determines the local stability of every equilibrium.

**Evaluation at the boundary equilibrium $\boldsymbol{x = 0}$**

Substituting $x = 0$,

$$J(0) = L - c - r.$$

Therefore, if

$$c + r > L,$$

then

$$J(0) < 0,$$

and the no-treatment equilibrium is locally stable.

Conversely, if

$$c + r < L,$$

then

$$J(0) > 0,$$

and the no-treatment equilibrium becomes unstable.

These boundary conditions establish the local stability of the no-treatment equilibrium analytically.

**Evaluation at the boundary equilibrium $x = 1$**

Substituting $x = 1$,

$$J(1) = -[p(k)L - c].$$

Since the coordination-game condition requires

$$p(k)L > c,$$

it follows that

$$J(1) < 0.$$

Thus, the full-treatment equilibrium is locally stable.

This confirms that when the population reaches universal treatment, small deviations do not move the system away from that equilibrium.

**Evaluation at the interior equilibrium**

An interior equilibrium exists whenever

$$L - c - r + x\left(r - L\big(1 - p(k)\big)\right) = 0.$$

Solving for $x$ gives

$$x^* = \frac{c + r - L}{r - L\big(1 - p(k)\big)}.$$

At this equilibrium,

$$G(x^*) = 0,$$

so the first term of the Jacobian disappears.

Consequently,

$$J(x^*) = x^*(1 - x^*)\big[r - L\big(1 - p(k)\big)\big].$$

Because

$$0 < x^* < 1,$$

and

$$r > L\big(1 - p(k)\big),$$

all three terms are positive.

Hence,

$$J(x^*) > 0.$$

Therefore, the interior equilibrium is locally unstable.

This unstable equilibrium acts as a threshold separating the basins of attraction of the two boundary equilibria. Initial treatment adoption below the threshold converges toward the no-treatment equilibrium, whereas adoption above the threshold converges toward universal treatment.
